# Tongue squamous cell carcinoma as a possible distinct entity in patients under 40 years old

**DOI:** 10.3892/ol.2014.2054

**Published:** 2014-04-09

**Authors:** QI-GEN FANG, SHUANG SHI, FA-YU LIU, CHANG-FU SUN

**Affiliations:** 1 Department of Oral and Maxillofacial Surgery, China Medical University, Shenyang, Liaoning 110002, P.R. China; 2 Department of Pediatric Dentistry, School of Stomatology, China Medical University, Shenyang, Liaoning 110002, P.R. China

**Keywords:** oral cancer, squamous cell carcinoma, young patients, oral tongue

## Abstract

Much controversy exists in the published literature regarding the clinical course and prognosis of tongue squamous cell carcinoma (SCC) in young patients. The aim of the current study was to evaluate the clinical results of tongue SCC in young patients. A total of 176 patients were included in this retrospective study. The patients were divided into two groups (young and old) according to an age cut-off of 40 years. The χ^2^ test and Kaplan-Meier method were used to analyze the variables. In total, 15 patients were <40 years old and placed into the young group, with five-year recurrence-free survival (RFS) and disease-specific survival (DSS) rates of 30 and 63%, respectively, compared with 47 and 62%, respectively, in the old group. No significant differences were identified between the RFS and DSS rates of the two groups, however, the young patients exhibited a different failure pattern. Overall, nine out of 10 recurrences in the young group occurred at a primary site compared with 18 out of 70 in the old group (P<0.001). Univariate analysis revealed that gender and differentiation were associated with recurrence and neck nodal involvement. In addition, poor differentiation was found to significantly decrease the DSS time. However, the prognosis of tongue SCC in the young patient group did not appear to differ from that of the old patient group. Furthermore, in the young patient group, local recurrence was the most common failure pattern and tumor differentiation was the most important prognostic factor.

## Introduction

Tongue squamous cell carcinoma (SCC) is the most common type of malignant tumor of the oral cavity, and usually occurs following the fifth decade of life. A previous study reported that <3% of these carcinomas develop in patients of <40 years old (1). In this subgroup of patients, the proportion of females was greater and a history of smoking and drinking was less frequent. A great deal of controversy exists regarding the clinical course and prognosis of tongue SCC between young and old patients in the published literature. Certain studies have reported that young patients exhibit improved clinical results when compared with old patients (2,3), whereas other studies have reported a significant decrease in the survival rate of young patient groups (4–6). However, further studies have revealed a similar prognosis between young and old patient groups (7–16).

In the present study, young and old patients with tongue SCC were compared to analyze disease recurrence and survival rates.

## Materials and methods

### Patient presentation

Approval for the current study was obtained from the Institutional Research Committee of the China Medical University (Shenyang, China). Between 2005 and 2011, 216 patients were treated for tongue SCC at the Oral Maxillofacial Head and Neck Tumor Center of China Medical University (Shenyang, China), however, 40 of these patients were excluded from the present study, as they were lost to follow-up. Therefore, a total of 176 patient medical records were reviewed. The patient information, including demographic data, tobacco and alcohol consumption, tumor stage, node stage, differentiation, recurrence and survival, were collected, and the patients were divided into two groups (young and old) according to an age cut-off of 40 years. Written informed consent was obtained from all patients.

### Statistical analysis

The χ^2^ test was used to evaluate the significance of the variables and the Kaplan-Meier method was used to analyze the recurrence-free survival (RFS) and disease-specific survival (DSS) rates. Statistical analysis was conducted using SPSS version 13.0 (SPSS, Inc., Chicago, IL, USA) and P<0.05 was considered to indicate a statistically significant difference.

## Results

### Patient characteristics of the young group

The young group consisted of 15 patients (six male and nine female), with an average age of 34.0 years (range, 22–40 years). In total, 33.3% of the patients had a history of tobacco use and 13.3% of patients had a history of alcohol consumption. Of the 15 tumors, four (26.7%) were staged as T1, eight (53.3%) as T2 and three (20%) as T4. Furthermore, six tumors (40%) were well-differentiated, four tumors (26.7%) were moderately-differentiated and five tumors (33.3%) were poorly-differentiated. The mean follow-up time was 38.7 months (range, 8–96 months) (Table I).

### Patient characteristics of the old group

The old patient group consisted of 161 patients (113 male and 48 female), with an average age of 57.9 years (range, 42–94 years). In total, 67.7% of the patients had a history of tobacco use and 44.7% had a history of alcohol consumption. Of the 161 tumors, 25 (15.5%) were staged as T1, 87 (54.0%) as T2, 16 (9.9%) as T3 and 33 (20.5%) as T4. Furthermore, 85 tumors (52.8%)were well-differentiated, 36 (22.3%) were moderately-differentiated and 40 (24.8%) were poorly-differentiated. The mean follow-up time was 37.9 months (range, 6–96 months) (Table I).

### RFS rates

**Figure 1:**
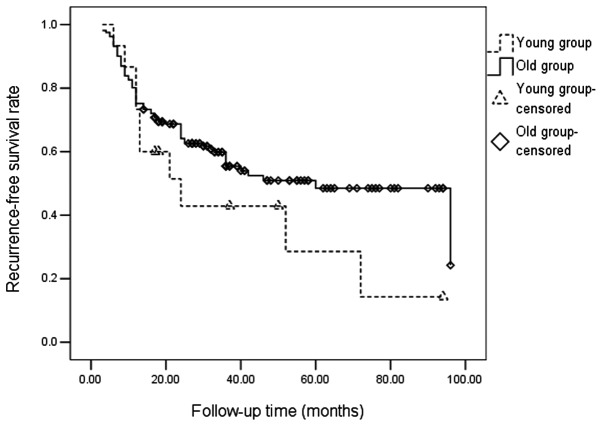
Comparison of recurrence-free survival (RFS) rates between the young and old patient groups (P=0.153).

Fig. 1 compares the RFS rates of the two groups. In the young group 10 patients (66.7%) exhibited locoregional recurrences (nine locally and one regionally) and the mean survival time until the first recurrence of the disease was 23.4 months (range, 6–72 months). In the old group, 58 patients (36.0%) exhibited locoregional recurrences (18 locally and 40 regionally) and 12 patients exhibited distant metastasis. Furthermore, the mean survival time until the first recurrence of the disease was 17.0 months (range, 3–96 months).

### DSS rates

**Figure 2:**
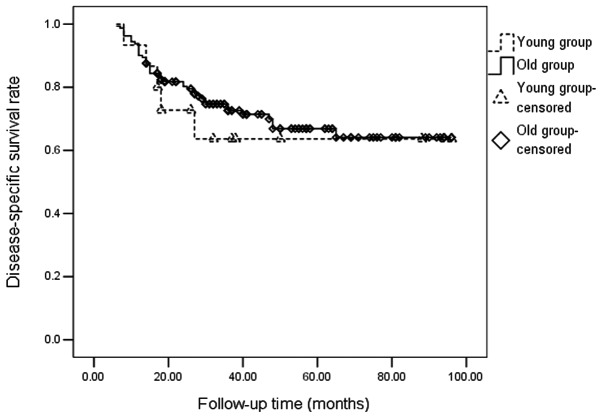
Comparison of disease-specific survival (DSS) rates between the young and old patient groups (P=0.631).

Fig. 2 compares the DSS rates of the two groups. In the young group, five patients succumbed to the disease and the mean survival time prior to mortality was 16.8 months (range, 8–27 months). In the old group, 44 patients (27.3%)succumbed to the disease and the mean survival time prior to mortality was 20 months (range, 7–65 months).

### Univariate analysis

Univariate analysis of the young group suggested correlation between RFS and gender (P=0.059) and significant correlation with tumor differentiation (P=0.016), whereas node metastasis (P=0.016) and tumor differentiation (P=0.041) were found to significantly correlate with DSS.

## Discussion

Traditionally, tongue SCC arises in middle-aged male patients following decades of tobacco and alcohol abuse, however, more recent studies have reported the increased incidence of tongue SCC in young individuals. Park *et al* (5) reported that 23 (27.1%) out of 85 patients with tongue SCC were <40 years old, while Liao *et al* (11) reported that 76 (25.8%) out of 296 patients with tongue SCC were <40 years old. However, the current study identified that the patients with tongue SCC who were <40 years old only presented 8.5% of the total patients.

Similar to the studies by Popovtzer *et al* (10), Friedlander *et al* (7) and Yip *et al* (14), the present study identified a higher percentage of females, non-smokers and non-drinkers in the young group compared with the old group. In addition, univariate analysis revealed that the female gender tended to be predictive of an improved RFS rate (P=0.059). However, Vargas *et al* (6) reported that young females with SCC of the anterior tongue exhibited significantly higher rates of recurrent disease.

Furthermore, consistent with the study by Pitman *et al* (13), the present study identified that the tumors of the young group appeared to be weakly aggressive at diagnosis; the majority of tumors (80%) were staged as T1–T2 and only one tumor exhibited positive node metastasis. However, the clinical result was poor, as 10 (66.7%) patients exhibited recurrence and five (33%) patients succumbed to the disease. In addition, the univariate analysis revealed that positive nodal metastasis was found to significantly correlate with a decreased DSS time (P=0.016). Manuel *et al* (9) also reported a similar observation in which the five-year DSS of patients with histologically-positive nodes was significantly shorter compared with that of patients with pathologically-negative nodes. Additionally, Myers *et al* (17) reported that node stage was found to significantly correlate with decreased survival.

In the current study, no difference was observed in the differentiation distribution between the two age groups (P=0.677), a result which has been confirmed by Park *et al* (5), but is contradicted by other studies. Veness *et al* (18) reported that the proportion of poorly-differentiated tumors in the young group was greater, however, Manuel *et al* (9) and Atula *et al* (15) reported that the majority of the tumors in their series were well-differentiated. In the present study, tumor differentiation was identified to be the most important prognostic factor for young patients and was found to significantly correlate with the RFS (P=0.016) and DSS (P=0.041) rates. Similarly, Siegelmann-Danieli *et al* (16) concluded that tumor pathogenesis tended to predict clinical course, and Atula *et al* (15) revealed that moderately- to poorly-differentiated carcinomas exhibited a poor prognosis.

No overall consensus concerning the difference in prognosis between young and old groups has been reached, however, more recent studies have indicated similar clinical results (3–10). Furthermore, the current study indicated that the prognosis of the young patients markedly resembled that of the old patients. However, a trend was identified for the young patients to exhibit an increased rate of locoregional recurrence (66.7% vs. 36.0%) and a decreased rate of distant metastasis (0 vs. 7.5%) compared with the old patients. Additionally, nine out of 10 recurrences in the young group occurred at a primary site compared with 18 out of 70 recurrences in the old group, and this difference was found to be significant (χ^2^ test; P<0.001). Local failure was more common in the young patients, and the possible causes were hypothesized to be due to the majority of tumors (60%) being moderately- or poorly-differentiated and the fact that only one tumor exhibited neck nodal involvement in the young patients. Pitman *et al* (13) and Garavello *et al* (4) found that local recurrence was more common in young patients, however, Pitman *et al* (13) also reported that regional failure and distant metastasis were similar among young patients and controls. Furthermore, Friedlander *et al* (7) revealed a trend toward increased primary and regional recurrence in the young population and Soudry *et al* (8) reported a higher percentage of distant failure in the young group compared with the old group.

Notably, the present study observed that failure at the primary site carried a poor prognosis in the young group. In total, five out of nine young patients with local recurrences succumbed to the disease and the majority of the mortalities (80%) occurred within two years of the initial surgery. Popovtzer *et al* (10) also revealed that young patients exhibit an aggressive course, with 40% mortality during the first two years.

In conclusion, the RFS and DSS rates identified in the current study were similar between the young and old groups, however, the failure pattern appeared to vary. The young patients exhibited an increased chance of local recurrence, and univariate analysis revealed that tumor differentiation was the most important prognostic factor for the young patients.

## Figures and Tables

**Table I:** Summary of patient information.

Parameters	Young group (n=15)	Old group (n=161)	P-value^a^
Age, years (range)	34.0 (22–40)	57.9 (42–94)	<0.001
Gender [n, (%)]			
Male	6 (40.0)	113 (70.2)	0.023
Female	9 (60.0)	48 (29.8)	
Habits [n, (%)]			
Tobacco use	5 (33.3)	109 (67.7)	0.008
Alcohol consumption	2 (13.3)	72 (44.7)	0.026
Tumor stage [n, (%)]			
T1	4 (26.7)	25 (15.5)	0.484
T2	8 (53.3)	87 (54.1)	
T3	0 (0.0)	16 (9.9)	
T4	3 (20.0)	33 (20.5)	
Node stage^b^ [n, (%)]			
N0	14 (93.3)	121 (75.2)	0.329
N1	1 (6.7)	26 (16.1)	
N2	0 (0.0)	13 (8.1)	
Differentiation [n, (%)]			
Well	6 (40.0)	85 (52.8)	0.677
Moderate	4 (26.7)	36 (22.4)	
Poor	5 (33.3)	40 (24.8)	
Treatment [n, (%)]			
Surgery	10 (66.7)	99 (61.5)	0.648
Surgery + radiation	5 (33.3)	50 (31.1)	
Surgery + radiation + chemotherapy	0 (0.0)	12 (7.5)	
Follow-up, months (range)	38.7 (8–96)	37.9 (6–96)	0.566

a χ^2^ test;

b Node stage of one patient in the old group was unknown.
